# Supplementary material for: Predicting visual function by interpreting a neuronal wiring diagram
Source: Nature. 2024 Oct 2;634(8032):113–23. doi: 10.1038/s41586-024-07953-5 (PMC11446822; doi:10.1038/s41586-024-07953-5)
Supplement: Supplementary file 6 — CRF and ERF predictions for individual TmY4 and TmY9 cells. Analogous to Supplementary Data 3, but for TmY target types. Shown are the top four monosynaptic pathways, the strongest pathway passing through each of the top ten intermediary types (ranking from Extended Data Fig. 7), and the trisynaptic pathway Tm1–TmY–Dm3–TmY (see the section entitled Prediction of spatial normalization). [file 41586_2024_7953_MOESM6_ESM.zip › DataS4/TmY9q/720575940625678266.pdf]

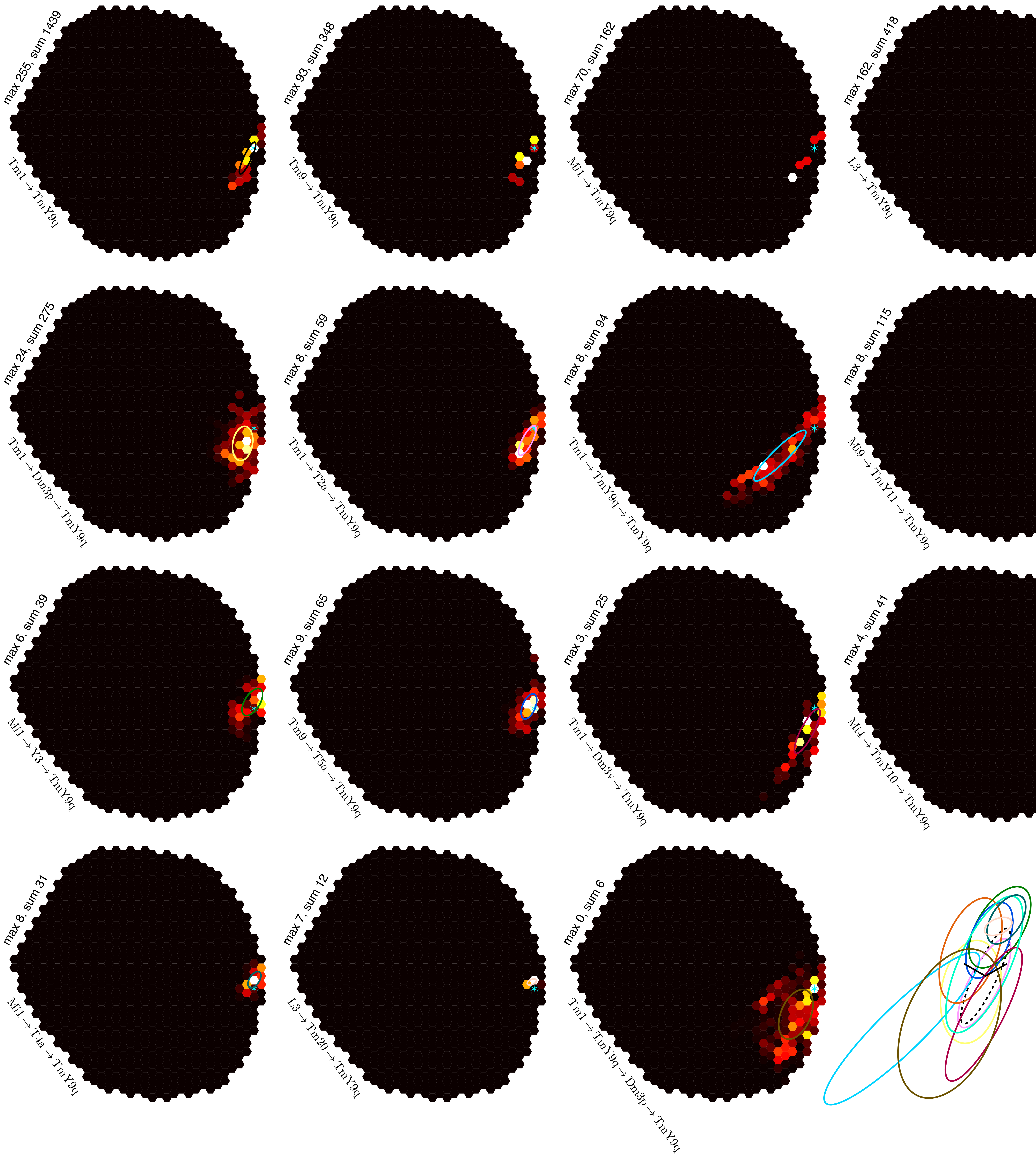
max 255, sum 1439
Tm1 → TmY9q
max 93, sum 348
Tm9 → TmY9q
max 70, sum 162
Mi1 → TmY9q
max 162, sum 418
L3 → TmY9q
max 24, sum 275
Tm1 → Dm3p → TmY9q
max 8, sum 59
Tm1 → T2a → TmY9q
max 8, sum 94
Tm1 → TmY9q → TmY9q
max 8, sum 115
Mi9 → TmY11 → TmY9q
max 6, sum 39
Mi1 → Y3 → TmY9q
max 9, sum 65
Tm9 → T5a → TmY9q
max 3, sum 25
Tm1 → Dm3v → TmY9q
max 4, sum 41
Mi4 → TmY10 → TmY9q
max 8, sum 31
Mi1 → T4a → TmY9q
max 7, sum 12
L3 → Tm20 → TmY9q
max 0, sum 6
Tm1 → TmY9q → Dm3p → TmY9q
